# Supplementary material for: Pkd2l1 is required for mechanoception in cerebrospinal fluid-contacting neurons and maintenance of spine curvature
Source: Nat Commun. 2018 Sep 18;9:3804. doi: 10.1038/s41467-018-06225-x (PMC6143598; doi:10.1038/s41467-018-06225-x)
Supplement: Supplementary file 1 — Supplementary Information [file 41467_2018_6225_MOESM1_ESM.pdf]

Supplementary Information

Title

Pkd2l1 is required for mechanoception in cerebrospinal fluid-contacting neurons and maintenance of spine curvature

Authors

Jenna R. Sternberg, Andrew E. Prendergast*, Lucie Brosse, Yasmine Cantaut-Belarif, Olivier Thouvenin, Adeline Orts-Del'Immagine, Laura Castillo, Lydia Djenoune, Shusaku Kurisu, Jonathan R. McDearmid, Pierre-Luc Bardet, Claude Boccara, Hitoshi Okamoto, Patrick Delmas, Claire Wyart*

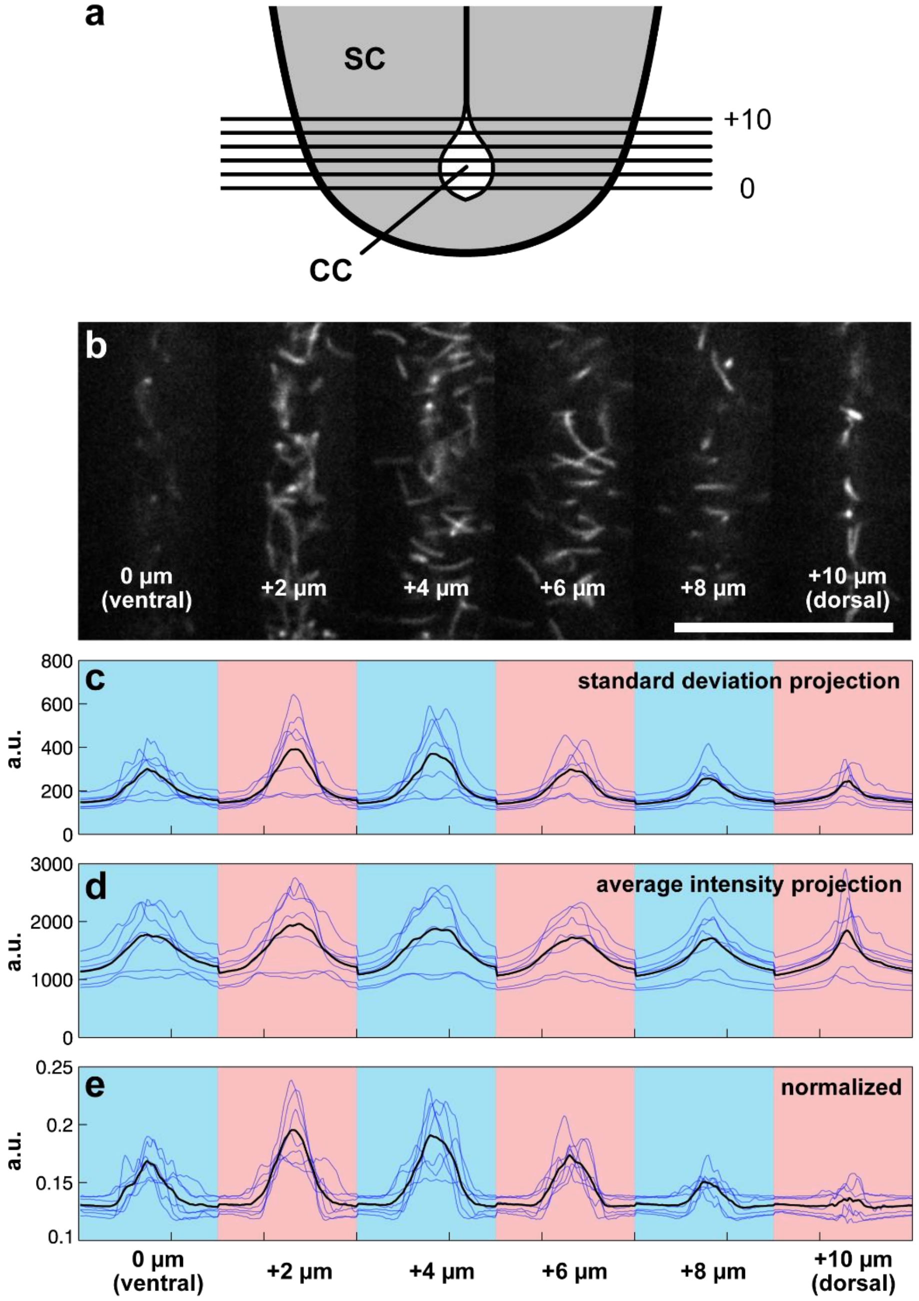

Supplementary Fig. 1. Estimation of wild type ciliary motion as a function of position in the dorsoventral axis.
(a) Schematic illustrating ciliary imaging experiments. Time-lapses are taken from optical slices covering the central canal moving ventral to dorsal 2 µm apart. (b) Representative frame from a conjoined time-lapse showing 6 planes of central canal cilia. Scale: 25 µm. (c) Intensity plot profiles derived from standard deviation projections. Blue traces are individual embryos, black trace represents average across all embryos. Blue and pink coloration defines the optical section. (d) Intensity plot profiles derived from average intensity projections. (e) Normalized profiles generated by dividing individual standard deviation profile plots by average intensity profile plots reveal a greater motility 2 - 4 µm away from the ventral boarder of the central canal. a.u. = arbitrary units.

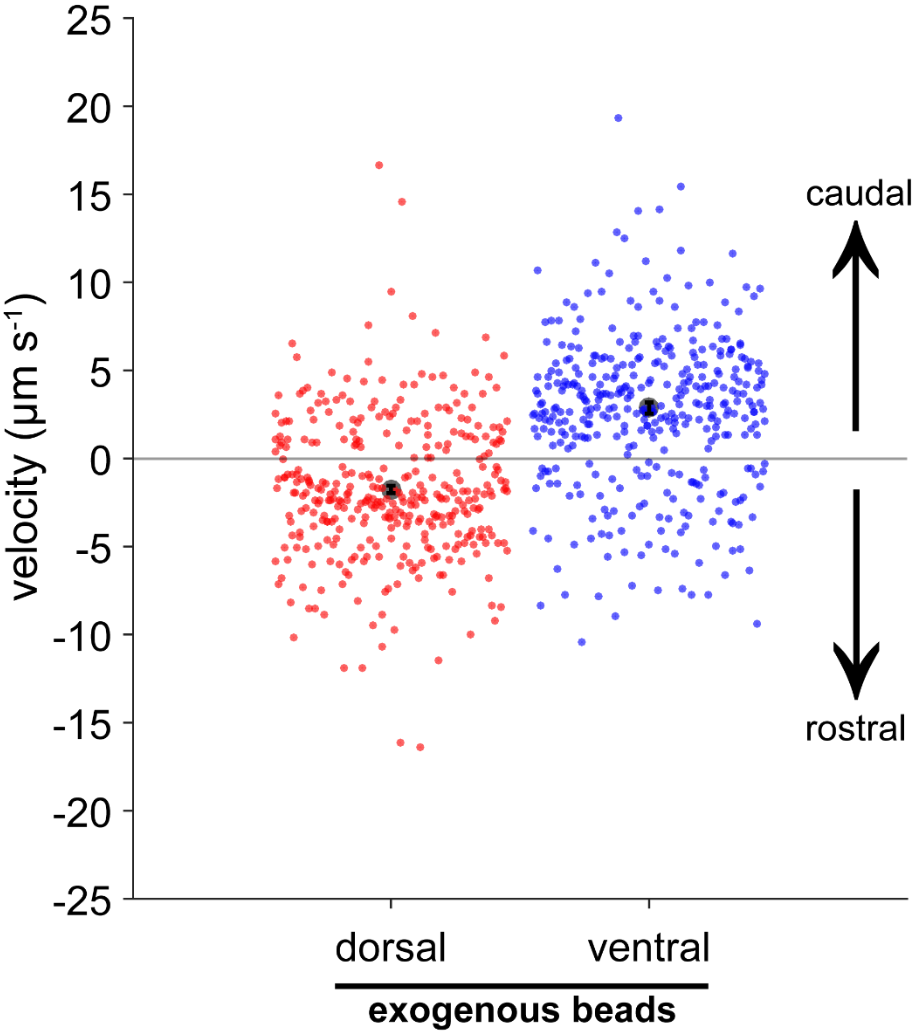

Supplementary Fig. 2. Bidirectional flow of the CSF in the central canal is preserved in the *pkd2l1*$^{-/-}$ mutant.

Each point represents one trajectory. Net dorsal movement is posterior to anterior; net ventral movement is from anterior to posterior. Dorsal mean velocity: 1.76 ± 0.21 µm s^{-1}; ventral mean velocity: 2.86 ± 0.32 µm s^{-1}. n = 362 dorsal trajectories, n = 381 ventral trajectories from n = 5 *pkd2l1*$^{-/-}$ embryos. Error bars represent s.e.m.

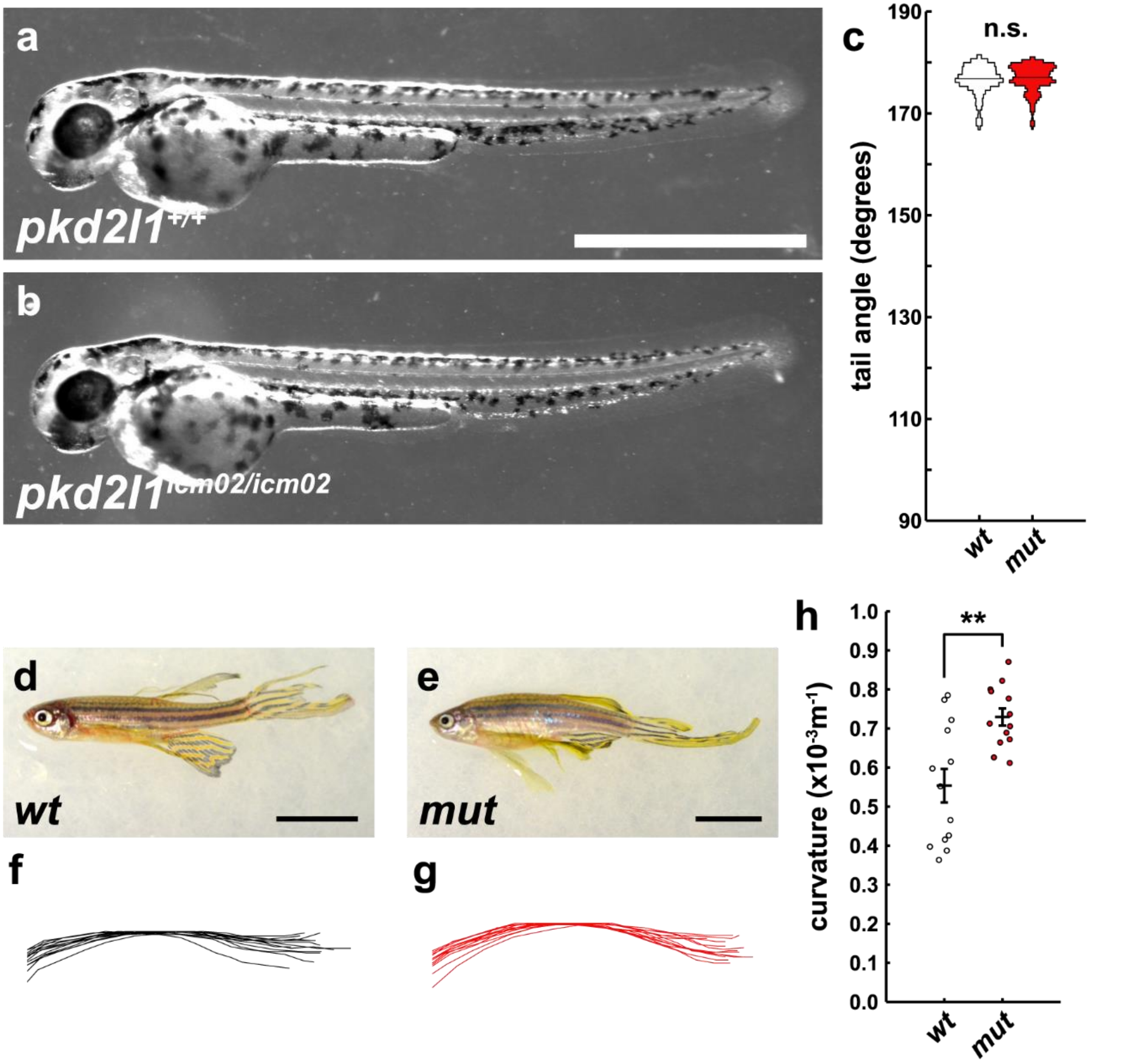

Supplementary Fig. 3. After three generations, *pkd2l1*$^{icm02/icm02}$ fish continue to develop kyphosis as adults.
(a) 48 hpf wild-type larva exhibiting normal development and a near 180° tail angle. Scale bar: 1 mm. (b) No difference is observed in the 48 hpf *pkd2l1*$^{icm02/icm02}$ larvae. (c) Violin plot of tail angles comparing a wild-type clutch to a mutant clutch at 48 hpf; there is no significant difference in tail angle. (d) Wild-type adult fish at 12 months. Scale bar: 1 cm. (e) *pkd2l1*$^{icm02/icm02}$ adult fish at same age. Scale bar: 1 cm. (f) Traces of spinal curvature derived from widefield images taken of wild-type adults. (g) Same traces from *pkd2l1*$^{icm02/icm02}$ mutant adults. (h) Quantitative comparison of spinal curvature between wild-type and mutant adults at 12 months. Mutant spinal curvature is significantly greater (p = 0.0013, two-sample t-test). Error bars represent s.e.m.

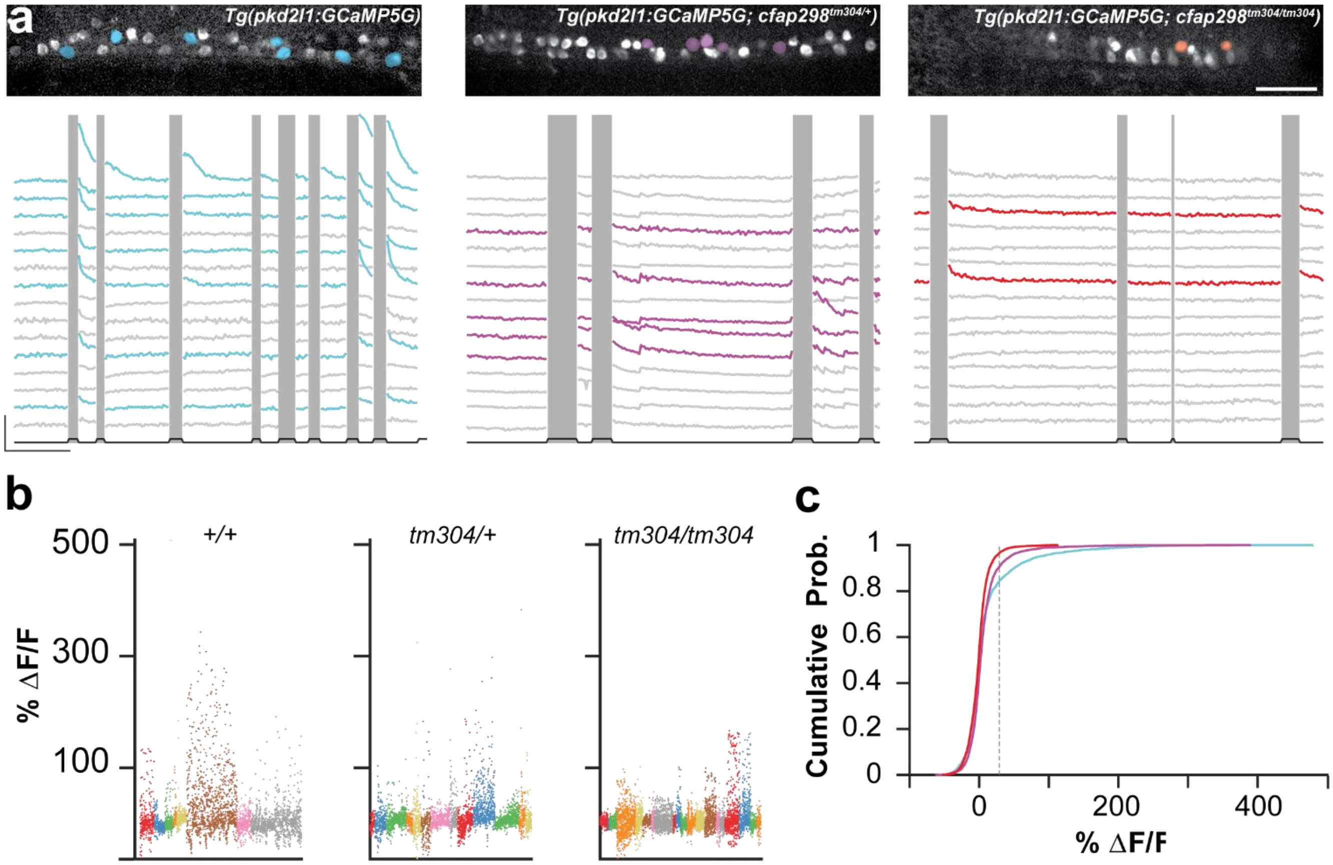

Supplementary Fig. 4. The *cfap298* mutation does not abolish sensory responses to muscle contraction in 1 dpf zebrafish.

(a) Top: Images of *Tg(pkd2l1:GCaMP5G)* in *cfap298*$^{+/+}$, *cfap298*$^{tm304/+}$, *cfap298*$^{tm304/tm304}$. Scale: 50 µm. Bottom: Sample calcium imaging traces of CSF-cNs at 1 dpf during active muscle contractions. Gray bars indicate periods of contraction when the cell moves out of the imaging plane. Scale: 10 s, 100% ΔF/F. (b) Responses to muscle contractions in individual cells for each genotype (*cfap298*$^{+/+}$ n = 7 fish, 140 cells; *cfap298*$^{tm304/+}$ n = 12 fish, 275 cells; *cfap298*$^{tm304/tm304}$ n = 20 fish, 430 cells). (c) Cumulative distribution plot of the responses for all genotypes shows a reduced response of CSF-cNs to spontaneous contraction in *cfap298*$^{tm304/tm304}$ embryos (linear mixed models, $p = 5 \times 10^{-4}$).

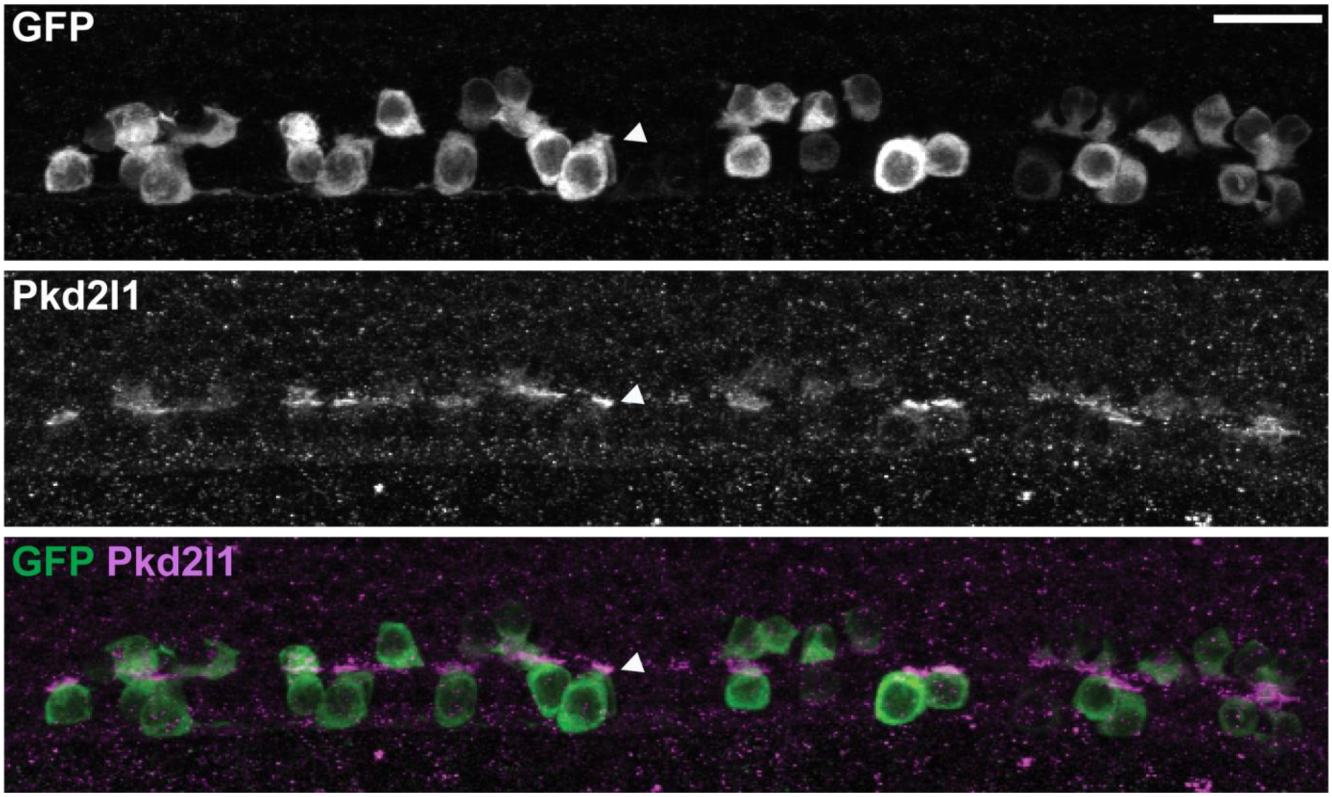

Supplementary Fig. 5. Pkd2l1 is correctly localized to the apical extension in the *cfap298*$^{m304/tm304}$ mutant.
Pkd2l1 immunohistochemistry in *Tg(pkd2l1:GCaMP5G; cfap298*$^{tm304/tm304}$*)* at 30 hpf. Top: GFP staining, middle: Pkd2l1 staining, bottom: merge. Arrowhead shows dense Pkd2l1 localization in the apical extension contacting the CSF in the central canal. Scale: 20 µm.

Supplementary Table 1. Statistics for Fig. 1a-b

		Imaging plane 1					
		0 µm	2 µm	4 µm	6 µm	8 µm	10 µm
Imaging plane 2							
	2 µm	0.0303					
	4 µm	0.0498	0.9999				
	6 µm	0.9976	0.0857	0.1323			
	8 µm	0.4127	0.0001	0.0003	0.1996		
	10 µm	0.079000	0.0	0.0	0.0277	0.9444	

Supplementary Table 2. Cobb angles for adult zebrafish

fish	ant. angle (°)	post. angle (°)	Cobb angle (°)
wild type 1	87.6	90	2.4
wild type 2	88.7	93.6	4.9
wild type 3	93.4	90.9	2.5
$pkd2l1^{-/-}$ 1	100.7	83	17.7
$pkd2l1^{-/-}$ 2	95.8	86.4	9.4
$pkd2l1^{-/-}$ 3	76.6	99.5	22.9

Supplementary Table 3. Zebrafish transgenic lines used in this study

Name used	Alternate name	Labeling	Original publication
$pkd2l1^{icm02}$	N/A	N/A	Böhm et al., 2016[20]
$cfap298^{tm304}$	$kurly^{tm304}$; $c21orf59^{tm304}$	N/A	Brand et al., 1996[10]
Tg(pkd2l1:GCaMP5G)	$Tg(pkd2l1{:}GCaMP5G)^{icm07}$	CSF-cNs	Böhm et al., 2016[20]
Tg(pkd2l1:gal4)	$Tg(pkd2l1{:}gal4)^{icm10}$	CSF-cNs	Fidelin et al., 2015[39]
Tg(UAS:TagRFP-CAAX)	$Tg(UAS{:}tagRFP\text{-}CAAX;cmcl2{:}eGFP)^{icm22}$	N/A	Böhm et al., 2016[20]
Tg(UAS:mCherry)	N/A	N/A	Herwig Baier Lab
Tg(β-actin:Arl13-GFP)	*Tg(actb2:Mmu.Arl13b-GFP)*	cilia	Borovina et al., 2010[15]
Tg(olig2:DsRed2)	N/A	olig2+ cells	Kucenas et al., 2008[45]
