# Supplementary material for: Pkd2l1 is required for mechanoception in cerebrospinal fluid-contacting neurons and maintenance of spine curvature
Source: Nat Commun. 2018 Sep 18;9:3804. doi: 10.1038/s41467-018-06225-x (PMC6143598; doi:10.1038/s41467-018-06225-x)
Supplement: Supplementary file 3 — Description of Additional Supplementary Files [file 41467_2018_6225_MOESM3_ESM.pdf]

Description of Additional Supplementary Files

File Name: **Supplementary Movie 1**
Description: **Cilia in a 24 hpf *Tg(β-actin:Arl13-GFP)* embryo.** Images were acquired at 33 Hz and are played in real time.

File Name: **Supplementary Movie 2**
Description: **Imaging of exogenous beads in the central canal of the spinal cord in a wild type embryo.** Images were acquired at 10 Hz and are played in real time.

File Name: **Supplementary Movie 3**
Description: **Imaging of endogenous particles with FF-OCT in the central canal of the spinal cord in a 26-28 hpf *Tg(β-actin:Arl13-GFP)* embryo.** Images were acquired at 100 Hz and are played in real time.

File Name: **Supplementary Movie 4**
Description: **Calcium imaging of CSF-cNs in a wild type *Tg(pkd2l1:GCaMP5G)* embryo at 24-26 hpf.** Images were acquired at 4 Hz and are sped up 5x in the movie.

File Name: **Supplementary Movie 5**
Description: **Calcium imaging in CSF-cNs in a $cfap298^{m304/tm304}$ *Tg(pkd2l1:GCaMP5G)* embryos at 24-26 hpf.** Images were acquired at 4 Hz and are sped up 5x in the movie.

File Name: **Supplementary Movie 6**
Description: **Calcium imaging of CSF-cNs in a $pkdl21^{-/-}$ *Tg(pkd2l1:GCaMP5G)* embryo at 24-26 hpf.** Images were acquired at 4 Hz and are sped up 5x in the movie.
